# The physical activity patterns of children with autism

**DOI:** 10.1186/1756-0500-4-422

**Published:** 2011-10-18

**Authors:** Megan MacDonald, Phil Esposito, Dale Ulrich

**Affiliations:** 1 School of Biological & Population Health Sciences, Exercise & Sport Science Program, Oregon State University, Corvallis, OR, USA; 2 Center on Physical Activity & Health in Pediatric Disabilities, School of Kinesiology, University of Michigan, Ann Arbor, MI, USA

## Abstract

**Background:**

Although motor deficits are gaining attention in autism research much less attention has been paid to the physical activity patterns in this group of children. The participants in this study were a group of children with autism spectrum disorder (N = 72) between the ages of 9-18 years. This cross-sectional study explored the physical activity patterns of seventy-two children with autism spectrum disorder as they aged.

**Findings:**

Results indicated significant differences between the mean time spent in moderate to vigorous physical activity and the mean time spent in sedentary activity. Older children with autism spectrum disorder are significantly more physically inactive, compared to younger children.

**Conclusions:**

Physical activity programs and interventions need to address this deficit, in physical activity. Children with autism have a similar trend in physical activity patterns compared to their peers without autism; associated benefits and future research will be discussed.

## Background

Autism spectrum disorder (autism) is a pervasive developmental disorder characterized by deficits in social skills, communication and repetitive or restricted interests [1]. Research has established that symptoms of movement disturbance are also present [2,3]. Although motor skill difficulties have started to receive more attention in autism literature, physical activity patterns have received less. Regrettably children with autism have not been spared from the obesity epidemic sweeping the United States [4,5]. Disturbing statistics suggest that children with autism are 40% more likely to be overweight and obese compared to their typically developed peers [4]. Increasing physical activity is a primary health objective in the United States [5]. Research has just started to explore the physical activity patterns of children with autism [6-8]. With that said the paucity of research in this area, combined with limitations to previous studies, makes it difficult to draw conclusions about physical activity patterns and associated health outcomes.

Nevertheless, even amidst the lack of physical activity and autism literature, one clear result is the positive influence of physical activity [9]. Following bouts of physical activity, children with autism experienced decreases in negative behavior such as stereotypies and increased positive behaviors, such as time on task [10]. More vigorous bouts of physical activity have further amplified positive behavior change in comparison to bouts of light or moderate physical activity [9,10]. Structured programs, for instance physical education, appear to foster more physical activity compared to unstructured environments, such as recess [8]. Pan and Frey (2006) found that children with autism had a similar trajectory of physical activity across age compared to typically developing children; unfortunately this indicated that physical inactivity became more prominent with increased age.

Over the past few years research in both physical activity and autism has been rigorous, leading to more advanced methods of assessment and sampling. Best practice recommendations for objective physical activity monitoring suggest the use of accelerometry consisting of at least 10 hours of monitor wear per day [11]. Previous objective physical activity research in children with autism was limited to 8 hours of accelerometer monitoring per day [7]. Since Pan & Frey's (2006) publication, research in physical activity has progressed beyond studying patterns of moderate to vigorous physical activity and more interest has begun to emerge in measuring sedentary physical activity patterns [12]. Finally, autism research has focused on sampling strategies inclusive of the high incidence of intellectual disability which exists within autism rather than sampling only 'high functioning' individuals [13].

The purpose of the present study is to describe both the sedentary and moderate to vigorous physical activity patterns of a cross-sectional sample of children aged 9-18 years with autism spectrum disorder as they age.

## Method

The Institutional Review Board at the University of Michigan approved all methods and procedures for this study. Seventy-two children with autism between the ages of 9-18 years (n = 72, male = 55, female = 17), who were recruited as a part of an adapted physical activity intervention study (teaching children how to ride a two-wheel bicycle), met the requirements to be included in this study. All participants were unable to ride a two-wheel bicycle prior to the study. An adapted two-wheel bicycle was used to teach the participants how to ride a conventional two-wheel bicycle. Parental consent and participant assent was obtained for all participants. To be included in this study all participants met autism cut-off criteria based on the mild-moderate and severe codes of the Social Responsiveness Scale (SRS), a valid assessment reliable in measuring autism severity [14,15]. Additionally all children met physical activity monitoring guidelines based on best practice recommendations (outlined below) [11,16].

### Physical Activity Measurement

Physical activity was measured using the Actical^® ^accelerometer (Mini Mitter/Respironics, Inc., Bend, OR) over a seven-day period during a typical week and prior to the adapted physical activity intervention. Data was collected during the spring months while the participants were still in school. The Actical^® ^accelerometer is one of the smallest accelerometers available (28 × 27 × 10 millimeters and 17 grams) and uses an omni-directional sensor with a 0.5-3 Hz range capable of detecting movements in all planes to create a composite measure of movement. The voltage generated by the sensor is amplified and filtered via analog circuitry and then passed into an analog to a digital converter, and the process is repeated 32 times each second (32 Hz). The resulting 1-second value is divided by four and then added to an accumulated activity value for the duration of the specified 15-second epoch [17]. For this study, a 15-second epoch was selected based on literature related to the erratic and sudden bursts of activity common to youth [17,18].

Participants wore the monitor for all waking hours of the day on the right ankle using an elastic belt. The monitor was to be worn for all activities except swimming, showering/bathing and sleeping. Parents/guardians of the participants were provided with a log to record any times when the monitor was not worn (i.e. forgetting to put it on in the morning, taking it off for comfort or any other reasons for which it may have been removed). Monitors were returned after a seven-day period via priority mail and were downloaded using an Actical Reader interface unit and associated software.

### Physical activity data reduction

Participants were included in this study if their physical activity data met the following criteria: the accelerometer was worn for at least four days (inclusive of at least one weekend day) for a minimum of 10 hours each day. These criteria have been previously established in the literature as suggested guidelines for obtaining valid and reliable accelerometry data [11,16]. Based on a 15-second epoch the data were then reduced and assigned to one of the following categories: sedentary activity (counts of <25), moderate physical activity (counts of 376-1625) or vigorous activity (counts > 1626). Data counts assigned to physical activity categories are related to energy expenditure validated in typically developing children [16,17].

Based on the time of day, physical activity data was partitioned by time spent in school, after school and evening hours. Time in school was between the hours 8:00 am - 3:00 pm, after school hours was between the hours 3:00 - 5:00 pm and evening hours were between the hours 5:00pm - 12:00am.

### Psychometric Measures

The Weshsler Abbreviated Scale of Intelligence (WASI) is a standardized measure of intelligence for individuals aged 6-89 years [19]. Two subtests were administered (the vocabulary and matrix reasoning subtests), to assess verbal and non-verbal intelligence and generate a standardized full-scale IQ. The WASI was administered to the participants by a clinician or graduate student with experience in cognitive assessment.

The SRS is a 65-item questionnaire, completed by the parent or guardian, and is valid and reliable in measuring autistic traits [14]. The SRS measures five areas of social development, as indicated by the parent: social awareness, social information processing, capacity for reciprocal social communication, social anxiety or avoidance and autistic traits. Furthermore the SRS provides a standardized score that qualitatively identifies the severity of autism ranging from mild to severe. Standard scores measured at 76 or higher represent a diagnosis in the severe range, standard scores measured between 60-75 resulted in a mild to moderate diagnosis.

### Anthropometric measures

Height and weight were measured without shoes. Height was measured in centimeters to the nearest tenth of a millimeter with a portable stadiometer (SECA S-214 portable stadiometer). Two measurement trials were administered and the average of the trials was recorded. Weight was measured in kilograms to the nearest gram (Health O Meter H-349KL digital scale). Two measurement trials were administered and the average of the trials was recorded. Body mass index (BMI) was calculated using the standard formula: body mass (kg.) divided by height (m^2^). Percentage of body fat was calculated using a gender-specific regression equation for children with triceps and calf skinfolds [20]. A physician experienced in measuring skinfolds, using Lange skinfold calipers, took two skinfold thicknesses at each site (triceps and calf) on the right side of the body. Measurements were taken twice at each site and rounded to the nearest tenth of a millimeter. The average at each site was used in the analysis.

### Data Analysis

All analyses were conducted in PASW version 18.0. Participants were initially divided into three age groups (9-11 years, 12-13 years & 14-18 years), however no significant differences were found between the 12-13 year age group and the 14-18 year age group, these age groups were combined for further analysis. Physical activity patterns were examined for each group in the sedentary and moderate to vigorous categories. Moderate and vigorous physical activity was combined for analysis based on established norms and recommendations in physical activity literature as the target intensity for receiving health benefits [21].

## Results

Eighty-two participants met the requirements for this study based on the physical activity reduction requirements and autism diagnosis per parental report. However, ten children were dropped from analyses based on an incomplete SRS or a score on the SRS falling within the normal range. A total of seventy-two children with autism met the requirements to be included in this study. Descriptive characteristics, of the children in this cross-sectional analysis are presented in table 1.

**Table 1:** Descriptive characteristics

	Age group 1	Age group 2
	N = 42	N = 30

WASI	*82.92 (± 19.69)	79.88 (±20.45)
SRS	Mild to moderate = 9 severe = 33	Mild to moderate = 11, severe = 19
Gender	**M = 34 F = 8	M = 21 F = 9
Race	***AA = 2, H = 1, C = 33, unspecified = 6	AA- 2, C = 22, A = 2, unspecified- 4
Height (cm)	141.7 (±10.90)	158.9 (±9.29)
Weight (kg)	39.94(±14.66)	60.46(±21.16)
BMI	19.42 (±4.32)	23.50 (±6.03)
BMI%ile	63.81(±29.96)	66.17(±32.9)
Waist circumference (cm)	69.29(±13.47)	82.77 (±17.96)

* Mean (standard deviation); ** M = male, F = female; ***AA = African American, H = Hispanic, C = Caucasian, A = Asian American

Preliminary analysis revealed that there were no significant differences in physical activity based on IQ, autism severity or gender. As a result gender was combined for the subsequent analysis. Given that participants wore the Actical monitor for different amounts of total time, we used analysis of covariance (ANCOVA) with the time spent wearing the monitor as the co-variate. The mean time spent wearing the monitor differed by time of day (daily total 17.6 hours; in school total 5.3 hours; after school total 1.9 hours; evening 5.1 hours).

ANCOVA of moderate to vigorous physical activity patterns revealed significant differences in the total (p < 0.05), in school (p < 0.01), after school (p ≤ 0.001) and evening (p < 0.05) physical activity patterns (see table 2). An estimate of the effect size, based on the partial Eta squared, can be found in table 2.

**Table 2:** Mean time spent in physical activity (based on daily average in minutes)

	Age group 1	Age group 2	P value	Partial ETA
	N = 42	N = 30		

Total time sedentary	666.67 (±107.17)	789.16 (±113.51)	≤0.001	0.36
In school sedentary	178.98 (±33.39)	218.38 (±44.09)	<0.001	0.50
After school sedentary	63.47(±15.38)	75.30 (±12.27)	<0.001	0.28
Evening sedentary	186.51 (±38.41)	221.98 (±50.11)	<0.05	0.40
Total moderate to vigorous	131.57(±84.23)	90.02 (±97.89)	< 0.05	0.07
In school moderate to vigorous	48.23(±21.90)	35.10 (±17.93)	<0.01	0.10
After school moderate to vigorous	17.32(±8.77)	10.28(±7.07)	≤0.001	0.16
Evening moderate to vigorous	40.48(±30.64)	25.99(±33.16)	<0.001	0.09

ANCOVA of sedentary physical activity patterns revealed significant differences in total (p ≤ 0.001), in school (p < 0.001), after school (p < 0.001) and evening (p ≤ 0.001) physical activity patterns. An estimate of the effect size, based on the partial Eta squared, can be found in table 2.

## Discussion

The results of this study clearly show declines in physical activity as children with autism age. This pattern is evident in decreased moderate to vigorous physical activity patterns as well as increased patterns of sedentary physical activity. Children with autism in this sample appear to be meeting the minimum requirements of physical activity. The observed age-related declines shed light on the lack of physical activity demonstrated in older children with autism. More specifically meaningful differences were found in moderate to vigorous physical activity patterns after school, similar to Pan and Frey's (2006) study, these results highlight a need for extracurricular after school programs. Although the youngest age group was found to be significantly more active in the immediate hours following school, small amounts of moderate to vigorous physical activity were obtained during the after school time frame, programs that incorporate longer bouts of physical activity also need to be explored.

The mean amount of time spent in moderate to vigorous physical activity after school for the youngest group was 17 minutes, followed by 10 minutes for the older age group. Unfortunately only a very small portion of time was spent in vigorous physical activity (approximately one minute for both age groups), this alone is reason for concern. In addition to the health-related benefits of physical activity research indicates that decreased stereotypy and self-stimulating behavior are the most common behavioral improvement following physical activity for children with autism and more pronounced effects were evident following vigorous bouts of physical activity [9,22]. Designing programs to increase vigorous bouts of physical activity in a naturalistic environment are critical to obtaining the positive benefits that have been derived from contrived exercise settings.

Without doubt, physical activity interventions for children with autism consistently show positive outcomes but the evidence is limited, which makes it hard to draw conclusions. In a recent review of physical activity interventions, a total of 18 studies were found, collectively only 64 participants received a physical activity intervention (across all 18 studies) [9]. The combination of limited descriptive and intervention-based studies in physical activity, along with an astounding rate of obesity in children with autism clearly displays a gap in the literature and a need to address such critical deficits.

This study shows age related declines in physical activity in children with autism. Nevertheless, based on current physical activity guidelines all age groups are meeting the minimum recommendation of 60 minutes of daily moderate to vigorous physical activity [5]. Although these recommendations have been met, we also know that proportionately, the majority of this time is spent in moderate physical activity (and not vigorous physical activity). At the same time, 43% of the children in this study are overweight (falling within the 85^th ^percentile based on gender and age). These contradicting factors, time spent in moderate to vigorous physical activity and BMI percentile, raise concerns about the current recommended guidelines for physical activity in this group of children, and whether or not the guidelines are in fact strict enough to see health related benefits. This relationship needs to be explored further and more rigorously.

Autism prevalence is at an all time high [23], and up-to-date research is at our fingertips. Although we are learning more about autism each day, descriptive research needs to be supported with intervention intended for change. Within a typically developing population of children physical activity programs have shown both social and physical health related benefits. Community-based physical activity programs may be a cost-effective alternative to clinical social skills programs while simultaneously addressing age-related declines in physical activity, obesity prevalence, movement deficits, friendship opportunities and social-communication practice in a natural setting. Although the benefits of physical activity are still being explored, the consensus is that everyone will benefit from a prescription of physical activity.

## Competing interests

The authors declare that they have no competing interests.

## Authors' contributions

MM conceived of the study, and participated in its design and coordination and drafted the manuscript. PE & DU participated in data collection, analysis and editing of the final manuscript. All authors read and approved the final manuscript.
